# Different Alginate Spherification Methods for Producing Hydrogel Beads Loaded with Vitamins B9 and B12 to Support Hydration in Older Adults with Dysphagia

**DOI:** 10.3390/gels12080706

**Published:** 2026-08-08

**Authors:** Enrika Lazickaitė, Milda Keršienė, Ina Jasutienė, Gytė Damulevičienė, Viktorija Eisinaitė, Daiva Leskauskaitė

**Affiliations:** 1 Department of Food Science and Technology, Kaunas University of Technology, LT-50254 Kaunas, Lithuania; lazickaite.enrika@gmail.com (E.L.); milda.kersiene@ktu.lt (M.K.); ina.jasutiene@ktu.lt (I.J.); daiva.leskauskaite@ktu.lt (D.L.); 2 Department of Geriatrics, Lithuanian University of Health Sciences, LT-44307 Kaunas, Lithuania; gyte.damuleviciene@lsmu.lt; 3 Geriatrics Center, Lithuanian University of Health Sciences, Kaunas Hospital, LT-47144 Kaunas, Lithuania

**Keywords:** dysphagia, encapsulation, alginate bead, hydration, spherification, food structure design

## Abstract

Adequate hydration and nutrition are crucial for preventing health complications and functional decline in older adults, especially those with dysphagia. This study examined the use of direct frozen and reverse spherification, with different residence times, to create alginate-based, high-water-content (>95%) bite-sized structures that are safe to swallow and capable of encapsulating vitamins B9 and B12 for delayed release in the gastrointestinal tract. Residence time notably affected the beads’ microstructure and texture. Reverse-spherified liquid-core beads were soft and stable, maintaining high water content; however, they were not suitable for people with dysphagia because of their uneven texture and water release when compressed or bitten. Conversely, direct frozen spherification yielded solid-core alginate beads with low hardness (77.46–136.25 g), minimal adhesiveness (~11 g·s), good cohesiveness (>0.3), and rheological properties compatible with swallowing. A fiberoptic endoscopic swallowing assessment found that the patients rated these solid-core beads as easy to chew and comfortable to swallow. Over six weeks, vitamin retention was 59.4–79.4% for vitamin B9 and 69.6–97.0% for vitamin B12, with 80–90% released during simulated digestion. These results highlight frozen direct spherification’s potential to create dysphagia-friendly, high-water-content finger foods that improve hydration, nutrition, and vitamin encapsulation.

## 1. Introduction

The global population is rapidly aging, posing significant challenges for healthcare systems worldwide [1]. As people age, they experience many physiological, sensory, psychological, and sociological changes that can affect their food intake and nutritional health [2,3]. Two key nutrition-related clinical issues in older adults are identifiable: one involves imbalances in nutritional components, including malnutrition, sarcopenia, and cachexia, with some overlap, and the other concerns fluid imbalances, such as dehydration and hypovolemia [4]. Providing older adults with adequate water and nutrients is essential to preventive efforts to reduce the risk of adverse health outcomes and physical decline [5].

An imbalance in nutritional components, particularly vitamins essential for certain organism functions. Some studies have indicated that adequate B vitamin intake is associated with reduced cognitive decline and a lower risk of all-cause dementia [6]. Water-soluble B vitamins must be obtained from the diet because the human body cannot synthesize them endogenously. Previous research indicated that low vitamin B12 intake is more commonly associated with Alzheimer’s disease [7], whereas insufficient vitamin B9 levels have been linked to cognitive decline and dementia [8].

Another key factor is low-intake dehydration, which is a water deficiency that occurs when beverage consumption does not meet fluid losses from urine, feces, respiration, and sweat [9]. It is observed in approximately 40% of patients aged 65 and older at the time of hospital admission [10]. Most water intake comes from drinking water (80%) and other beverages, with the remaining 20% from water in food [11]. This issue is particularly challenging for individuals with dysphagia, who often find it difficult or impossible to swallow liquids normally. Dysphagia is a prevalent syndrome among the elderly, characterized by difficulty in swallowing safely due to diminished muscle mobility and coordination [12], which can result in severe complications such as aspiration and suffocation [13]. This condition could markedly affect an individual’s health and quality of life [14].

Therefore, low-viscosity fluid foods should be thickened to slow the food bolus and facilitate safer swallowing. Adjusting the liquid’s texture typically involves adding food thickeners, but these have limitations. The functions of the thickeners can be affected by factors like temperature, duration, and pH level, with viscosity notably decreasing after 1–4 h [15]. Furthermore, some starch-based thickeners are susceptible to salivary amylase, which can reduce their thickness and increase pharyngeal residue [16]. The powder or paste form and flavor of these thickeners may also influence patient acceptance [17]. Another equally important observation is that older people, especially those with Alzheimer’s disease, often eat with their fingers, even when the food is unsuited to that—for example, puréed food. This demonstrates the main claim: so-called “finger foods” are necessary and beneficial for patients who want to eat independently and increase food intake [18].

Considering the circumstances outlined earlier, it is essential to develop new or improved liquid formulations that are safe and suitable for older adults and support their hydration as well as micronutrient needs. Gel-type products are particularly promising, as they can be designed for easy finger pickup, safe swallowing, high water content, and effective vitamin delivery. Past research has shown that hydrogel beads can increase water intake [19] and enable controlled vitamin release for patients with dysphagia [20]. These gel beads can also trap and transport various compounds within foods [21,22]. Beyond their functional role, they can improve texture and mouthfeel, making the products more appealing [23]. An evaluation of commercially available hydrogels intended for hydration in older adults indicates they are not specifically designed for patients with dysphagia. As a result, they may not be suitable for certain parts of older adults. Meanwhile, a large proportion of foods designed for dysphagia are not suitable for hydration and are consumed solely for their nutritional value.

Alginate is the most commonly used polysaccharide for making gel-type formulations. When cations are present, it can form two- or three-dimensional polymeric networks that are capable of holding large amounts of fluid [24]. Alginate is commonly employed in the food, medical, and pharmaceutical industries because of its safety, low cost, simple preparation, high biocompatibility, and biodegradability [25].

The internal and external structures of gel beads vary depending on their intended use. They can be produced as either solid- or liquid-core beads by modifying the spherification process, such as through direct or reverse spherification [23]. One study investigated alginate liquid-core hydrogel beads produced by reverse spherification to improve hydration in older adults. However, it applied only reverse spherification, and it did not address dysphagia, long-term vitamin storage, or vitamin release during digestion [19]. To the best of our knowledge, no studies have employed frozen direct alginate spherification for systems designed for hydration, vitamin delivery, and patients with dysphagia, which could be a completely novel approach. In our previous study, hydrogels were developed that possessed all the functional properties listed above; however, agar and carboxymethylcellulose were used in their production, implying a completely different stabilization mechanism [20].

This study aimed to develop bite-sized, high-water-content alginate-based hydrogel beads using frozen direct and reverse spherification techniques. It evaluated their potential for dysphagia diets and for target-site delivery of vitamins B9 and B12. In addition to comparing various spherification techniques for forming hydrogel beads, the study investigated how different residence times (the duration that alginate remains in contact with calcium ions in the gelation bath) influence physical and textural properties, swallowing-related rheology, encapsulation efficiency, storage stability, and delayed vitamin release during digestion.

Furthermore, the study assessed suitability for dysphagia diets through International Dysphagia Diet Standardization Initiative (IDDSI) tests and fiberoptic endoscopic evaluation of swallowing (FEES).

## 2. Results and Discussion

### 2.1. Characterization of the Alginate Hydrogel Beads Prepared by Direct and Reverse Spherification

#### 2.1.1. Appearance

**Figure 1:**
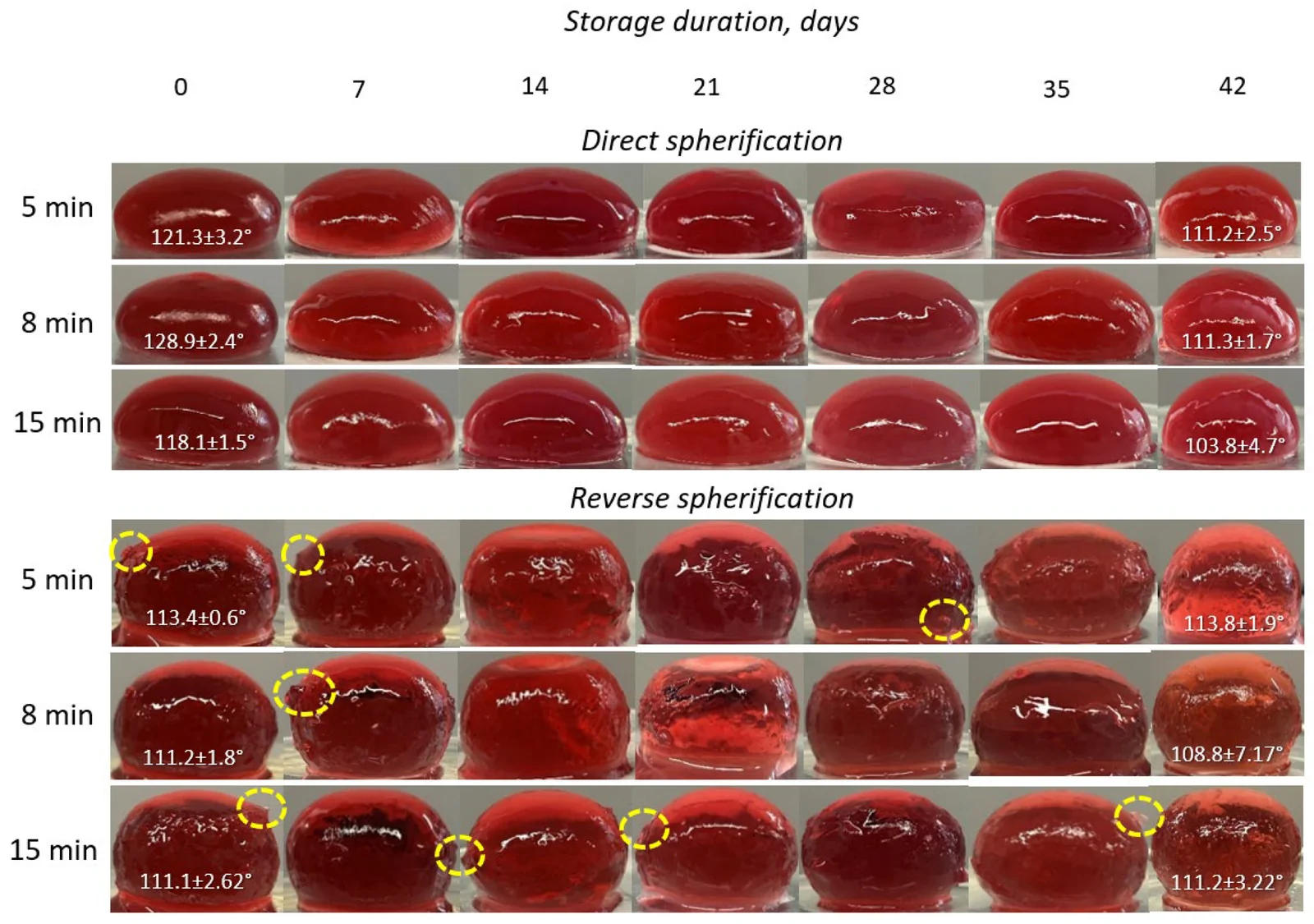
Visual appearance and contact angle of alginate hydrogel beads prepared by frozen direct and reverse spheroidization with different dipping times in the cross-linking solution, stored at +4 °C for 42 days (*n* = 3); The areas outlined in yellow indicate higher gel concentrations on the bead surface.

Typically, alginate beads are produced at sizes ranging from micrometers to millimeters. In this study, however, the beads were larger and bite-sized, with notable elasticity. In such a frozen spheroidization process, the size and shape of the hydrogel pucks/beads are strongly affected by the type of frozen mold used [26]. The obtained hydrogel beads are easy to hold with fingers and can be brought to the mouth without damaging the outer layer. The shape of the beads was heavily influenced by the spherification method used. Beads produced via direct spherification were solid-core and appeared more hemispherical, whereas those produced via reverse spherification were liquid-core and displayed more oval morphologies. Additionally, hydrogel beads from direct spherification had a smoother surface, whereas reverse spherification resulted in a rougher texture with surface cavities and visible gel deposits (Figure 1, highlighted in yellow). This difference in appearance is affected by the method of spherification. In direct spherification, a frozen sodium alginate solution is dipped into a calcium ion (Ca^2+^)-containing cross-linking agent. Conversely, in reverse spherification, the roles are reversed: the sodium alginate solution acts as the cross-linking agent, and a calcium ion-containing solution, in frozen form, is dipped into it [27]. As noted by other authors, a rough surface may result from increased alginate chain density. This higher density offers more carboxyl and hydroxyl groups for calcium ion binding, which enhances cross-linking [28]. Additionally, there was no visible difference in the beads’ sphericity among hydrogels with varying residence times (Figure 1), consistent with earlier studies [29].

The bright red color came from the cherry-apple juice concentrate in the recipe, which can be easily adjusted by changing the juice type while keeping the pH values the same. This adjustment is thought to increase the beads’ appeal to older patients, as the visual contrast between the bead and the plate may improve food and liquid intake, particularly in patients with Alzheimer’s disease [30].

#### 2.1.2. pH, Weight Loss, and Contact Angle

The pH of direct spherification hydrogel beads remained mostly stable over six weeks, staying within the acidic range of 3.7 to 4.0. At these low pH levels, alginate tends to form insoluble networks because its carboxyl groups are non-ionized, leading to denser structures with stronger chain interactions [31,32]. When pH reaches ≥5, the microstructure may become less interconnected and less dense, leading to syneresis and reduced hydrogel bead stability [32]. In hydrogel beads with a 5 min residence time, a slight increase in pH was observed during the first two weeks of storage. This change may have been due to the shorter interaction time with calcium ions, which prevented ionic bonds from fully forming. As the gel structure stabilizes during storage, some free carboxyl (-COOH) groups become ionized, leading to slightly stronger electrostatic repulsion and a rise in pH [31]. A similar pattern was observed in alginate beads produced by reverse spherification, with pH values changing only slightly and remaining below or at 4.1 throughout the storage period. Similarly, authors developed liquid-core alginate beads containing orange juice to enhance hydration in the elderly. They reported a pH of 4.16 and confirmed that the formulation is safe for use by older adults [19].

During the assessment of weight variations in alginate beads produced by direct spherification, hydrogel beads with the longest residence time (15 min) showed the greatest weight loss—25.24 ± 0.65% at the end of storage. Conversely, those with the shortest contact time (5 min) showed a weight loss of only 15.69 ± 1.37%. Both residence time and calcium ion concentration are critical factors influencing the water retention capacity of the hydrogel beads. An increased number of calcium ions binding to the alginate molecules results in greater cross-linking, which subsequently diminishes the hydrogel bead capacity to retain water molecules and consequently increases syneresis [33]. It has also been reported that a higher concentration of calcium ions (in our case, longer residence time) could increase the gelation velocity, which, in most cases, could lead to poorer uniformity of the obtained hydrogel beads [34]. It is also assumed that the hydrogel beads’ faster weight loss was due to their larger size (diameter ~15 mm) and greater surface area, both of which were significantly larger than those of the alginate beads produced by the conventional method (diameter ~5 mm) [35].

Weight differences arose from both the hydrogel beads’ manufacturing techniques and storage conditions. Specifically, alginate hydrogel beads produced via reverse spherification were stored in the preparation solution, thereby preventing moisture loss and facilitating mass exchange between the hydrogel beads and the storage medium via the interfacial film. Table 1 indicates that about 50% of the hydrogel beads’ total mass loss over 42 days—ranging from 4.27% to 6.66%—took place within the first 7 days of storage. This loss occurred as water permeated from the bead core across the alginate network membrane, consistent with findings from other studies using reverse spherification for plant-based yolk formulations [36]. However, as storage time increased, the weight of the alginate beads continued to decrease, indicating that slow water diffusion from the beads was driven by the osmotic pressure difference, which was more pronounced (*p* < 0.05) in the sample with a residence time of 8 min. This shows that the interfacial film formed during cross-linking could not effectively hinder moisture permeation during prolonged storage of the beads. This can be a significant challenge when encapsulating bioactive, low-molecular-weight compounds in this type of hydrogel beads.

The obtained weight loss results were also confirmed by changes in the sphericity of the formulated alginate beads, with a much smaller reduction in the contact angle in the beads made with reverse spherification.

#### 2.1.3. Texture

Textural properties are vital to oral processing and sensory perception, influencing consumer acceptance and swallowing safety in patients with dysphagia. This study highlights hardness, adhesiveness, and cohesiveness as the key properties of alginate beads.

In addition to its importance for patients with dysphagia, food hardness also affects the structural stability of hydrogel beads and their resistance to external pressure [37]. Regardless of the spherification method, alginate bead hardness increased progressively with an increase in residence time, indicating the formation of a stronger “egg-box” network of cross-linked guluronate blocks and impairing the mechanical strength of the hydrogel network [38], consistent with other studies [36]. Alginate bead hardness values remained low (<150 g), indicating that the beads are easily compressed by tongue force and can form a swallowable bolus. Previously reported alginate–xanthan gum liquid-core hydrogel beads had higher hardness values (222.35 g), most likely due to a longer residence time (16 min) applied during manufacturing [19]. All the formulated hydrogel beads were robust enough for manual handling, satisfying a key requirement for finger foods. However, the alginate bead with the shortest residence time should be handled carefully due to its relatively soft texture.

Given their structural properties, cohesiveness and adhesiveness were assessed only in alginate hydrogel beads produced through direct spherification. Cohesiveness, indicating the gel’s structural integrity, decreased from 0.35 to 0.29 with longer exposure to the cross-linking agent, a pattern that aligned with increased hardness (Table 2). All sample cohesiveness values complied with the Japan Modified Dietary Guidelines, which recommend that for people with dysphagia, the cohesion of “easy-swallowing food” should range from 0.2 to 0.9. This range helps prevent the food from breaking into smaller particles during swallowing [39]. The samples also showed very low adhesiveness (~11 g·s), suggesting limited ability of alginate beads to stick to oral or throat surfaces. Previous studies have indicated that foods with high adhesiveness might raise the risk of pharyngeal residue because of their stickiness, which could potentially cause aspiration during swallowing [40].

#### 2.1.4. IDDSI Tests

The texture analysis shows that the gels are soft, cohesive, and non-sticky, which could be beneficial for individuals with swallowing or chewing problems. However, to verify the suitability of the prepared hydrogel beads for a dysphagia diet, the International Dysphagia Diet Standardization Initiative (IDDSI) framework, which provides a standardized system for classifying dysphagia diets, was used. Fork pressure and separation tests were conducted.

**Figure 2:**
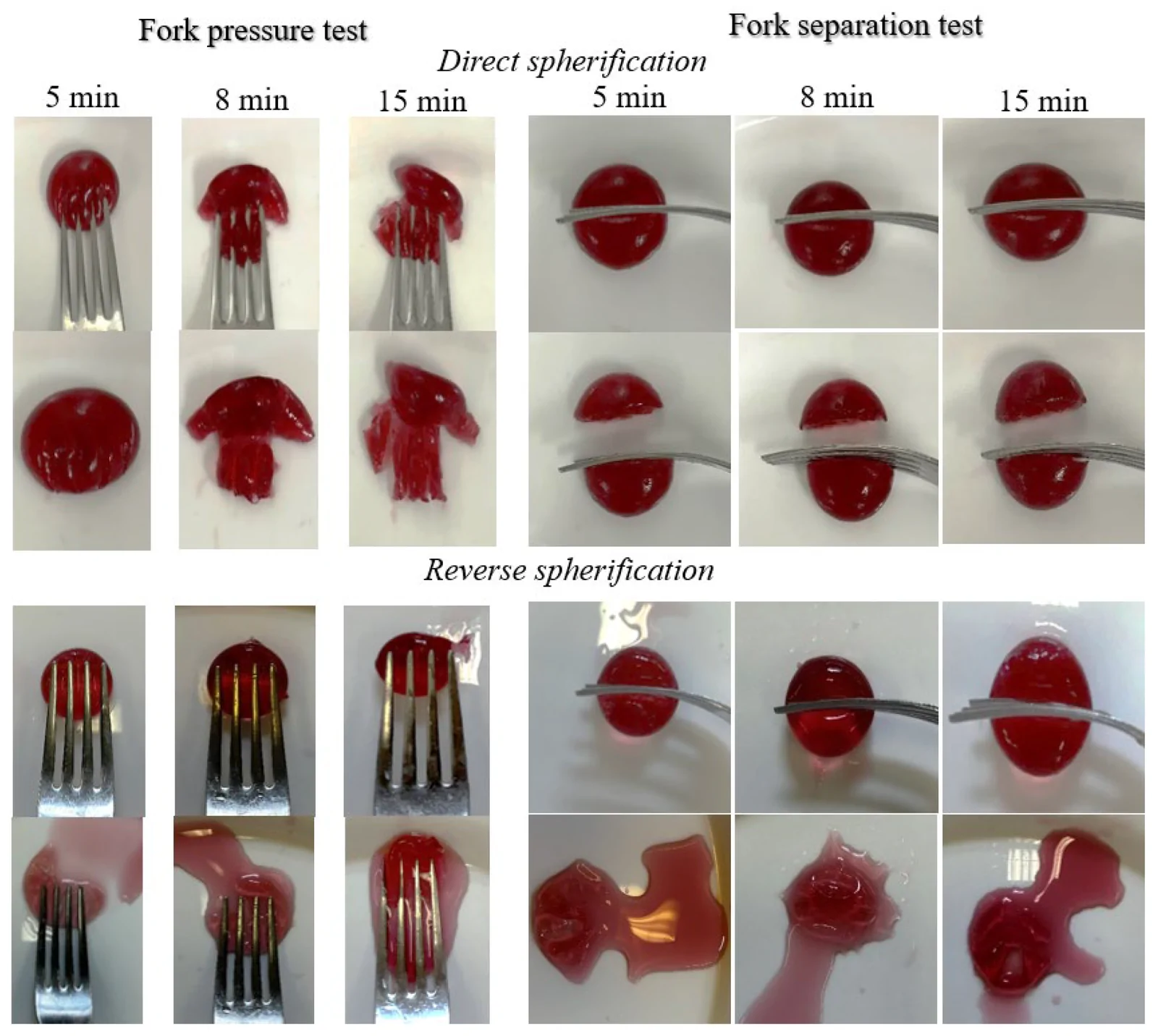
IDDSI test results for the alginate hydrogel beads prepared by frozen direct and reverse spherification at different residence times (*n* = 3).

As shown in Figure 2, beads formed via reverse spherification collapsed under applied force and released water. This behavior results from the spherification method, during which a gel layer forms on the surface of the frozen form, slowing calcium ion diffusion into the polymer and the initial solution. As a result, the liquid remains within without forming a gel, leading to a less uniform matrix with a weaker, less calcium-rich core and a more densely cross-linked shell [41]. This structural feature may result in a so-called “water burst” effect in the mouth and might be better suited to older adults without dysphagia who wish to minimize the sensation of thirst or dryness. However, for individuals with dysphagia, water released during biting could increase the risk of choking, which may be fatal. Compared to other forms, beads created through direct spherification have a denser internal structure (see Figure 2) and do not leak water when subjected to mechanical force. The fork compression test revealed that the hydrogel beads were crushed by fork prongs and did not return to their original shape after removal. According to the IDDSI classification, these beads are considered Level 6 soft, bite-sized foods. Importantly, after crushing, they did not break into many pieces, demonstrating strong internal bonds and ensuring safe swallowing.

### 2.2. Characterization of the Alginate Hydrogel Beads Prepared by Direct Spherification

Further studies were conducted exclusively with direct-spherification alginate beads to evaluate their safety for swallowing and their ability to deliver vitamins in a delayed manner. Beads produced via reverse spherification were deemed unsuitable not only due to the “water burst” effect but also because residual fragments of the alginate coating could pose a choking risk (Figure 2).

#### 2.2.1. Swallow-Related Rheological Properties

**Figure 3:**
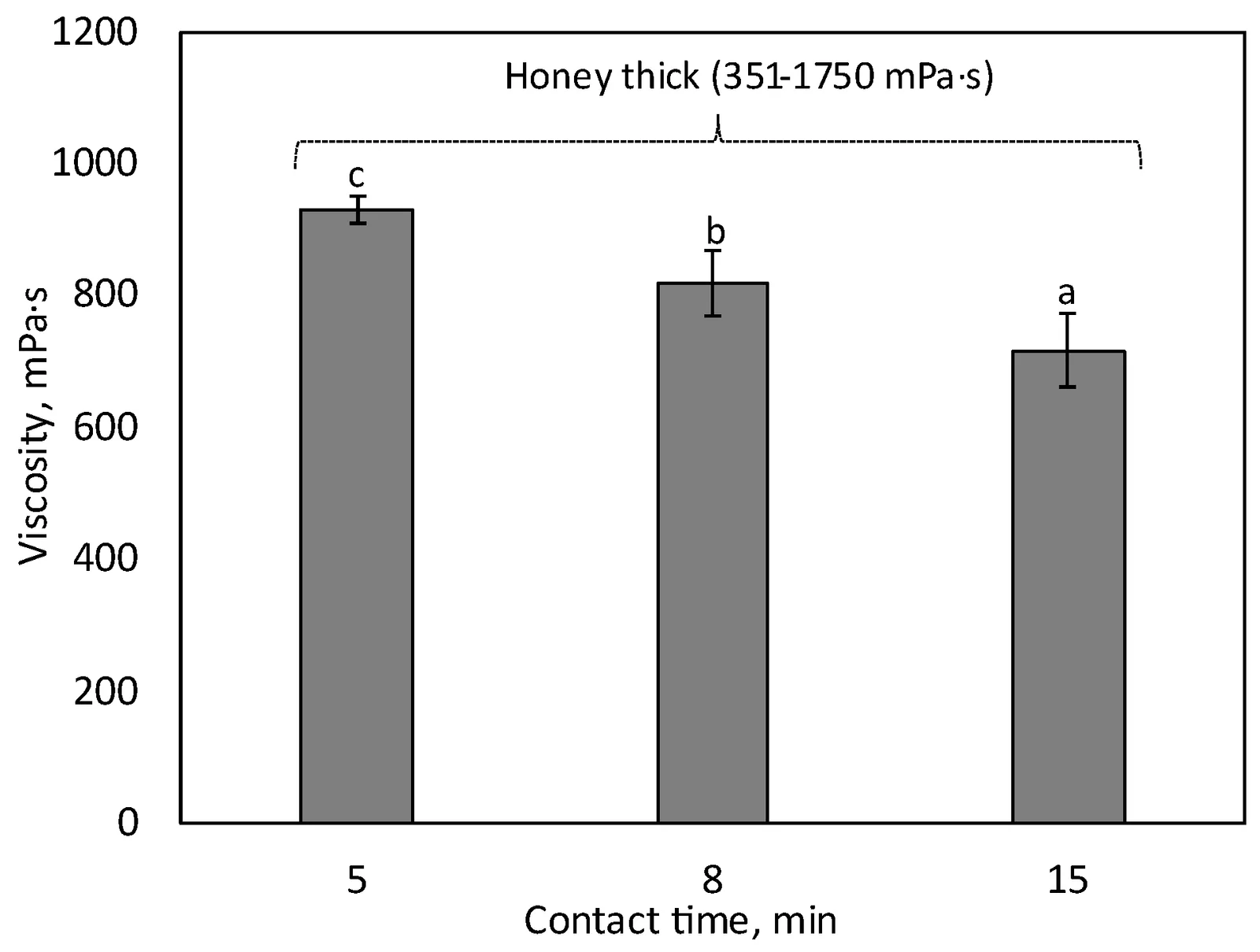
Viscosity at 50 s^−1^ for alginate hydrogel beads produced by frozen direct spheroidization with varying residence times (*n* = 3). (a–c) Different superscript letters represent significant (*p* < 0.05) differences in viscosity among alginate hydrogel beads with different residence times.

When measured at 50 s^−1^—resembling oral conditions—all formulated alginate beads had honey-thick viscosity levels (351–1750 mPa·s), with values between 716 and 930 mPa·s (Figure 3). These findings align with our previous research on hydrogel-based finger foods containing 0.8% agar [20]. It has been noted that foods with a viscosity under 5000 Pa·s at a shear rate of 50 s^−1^ are potentially suitable for individuals with swallowing difficulties [42].

**Figure 4:**
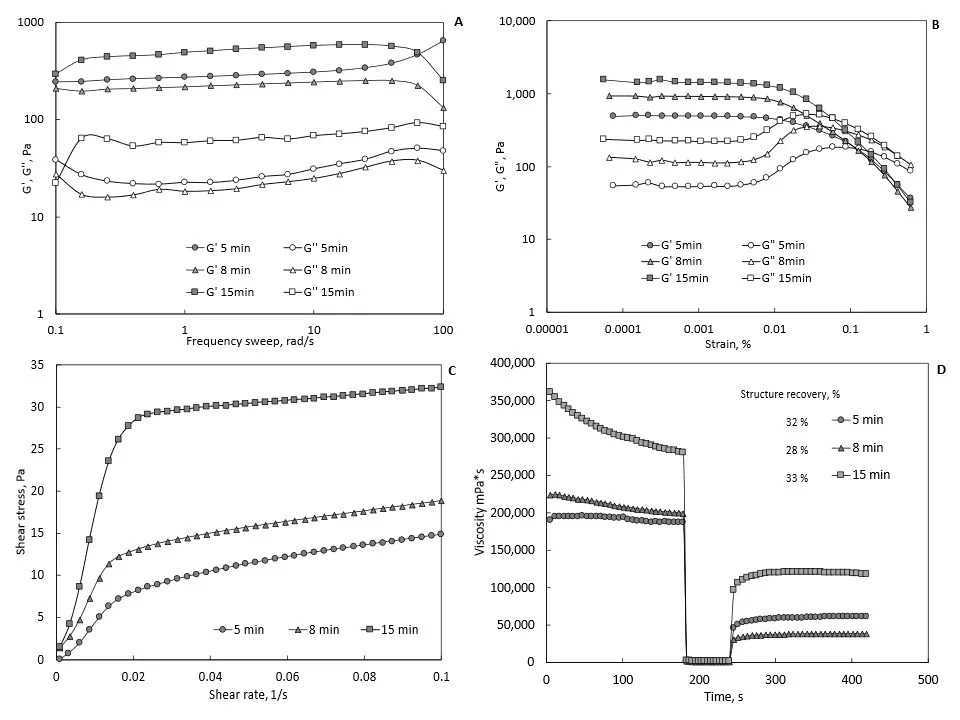
Frequency sweep (**A**); strain sweep (**B**); yield stress (**C**); and structure recovery (**D**) for alginate hydrogel beads produced by frozen direct spheroidization at varying residence times (*n* = 3); *p* < 0.05.

Oscillatory rheological tests assessed the viscoelastic properties of gel bead samples, vital for creating foods suitable for dysphagia. In the frequency sweep test, all samples showed a higher storage modulus compared to the loss modulus (Figure 4A), demonstrating gel-like behavior, similar to sodium alginate and monoglyceride bigel beads designed for dysphagia [43]. This trait is important for developing food products suitable for individuals with dysphagia [39]. Higher elastic modulus values at longer residence times indicate that prolonged contact between alginate and calcium ions improves the mechanical strength of alginate beads, as confirmed by the textural analysis. Regarding frequency dependence, it was clearly observed only at the highest applied frequencies, indicating the onset of structural deformation. Furthermore, all samples showed tan δ values (G″/G′) below 1.0 but above 0.1, indicating that the alginate beads exhibit weak gel properties. These features help the food bolus move smoothly through the pharynx and esophagus when swallowing.

Figure 4B shows that all samples exhibit strong gel strength at low strain levels. However, when the strain reaches the critical point, it indicates structural failure. These higher deformations resemble those experienced during oral processing, like tongue pressure or chewing [44]. Longer residence times strengthen the network, with the critical stress needed to break the bead network being twice as high—20.8 Pa—for beads with the longest contact time (15 min). Similarly, longer residence times led to higher yield stress values (29.2 Pa) (Figure 4C), which also correlated with increased hardness and critical stress. Higher critical and yield stresses may be considered unfavorable, as they indicate that greater tongue effort is required to compress the sample in the mouth, which could be problematic for patients with dysphagia [45].

The thixotropic recovery was tested under three shear-rate levels. Results indicated that at high shear rates, viscosity dropped sharply due to structural disruption. When exposed to low shear rate, the samples only partially recovered their original viscosity, reaching about 30% (Figure 4D). This low recovery rate is likely due to weak molecular interactions between alginate and calcium ions [42]. These changes are permanent and mimic what happens to the beads in the mouth. Structural recovery can help prevent the food bolus from refluxing into the oral cavity or entering the trachea, thereby improving swallowing safety [46].

The rheological results show that the hydrogel beads maintain a balance, neither becoming overly solid nor overly liquid. Reduced viscosity under shear improves swallowing while preserving sufficient structural integrity for stability. Based on all tests applied, it can be concluded that alginate hydrogel beads exhibit rheological properties compatible with swallowing dynamics.

#### 2.2.2. Swallowing Safety Evaluation by Fiberoptic Endoscopic Evaluation—Case Study

**Figure 5:**
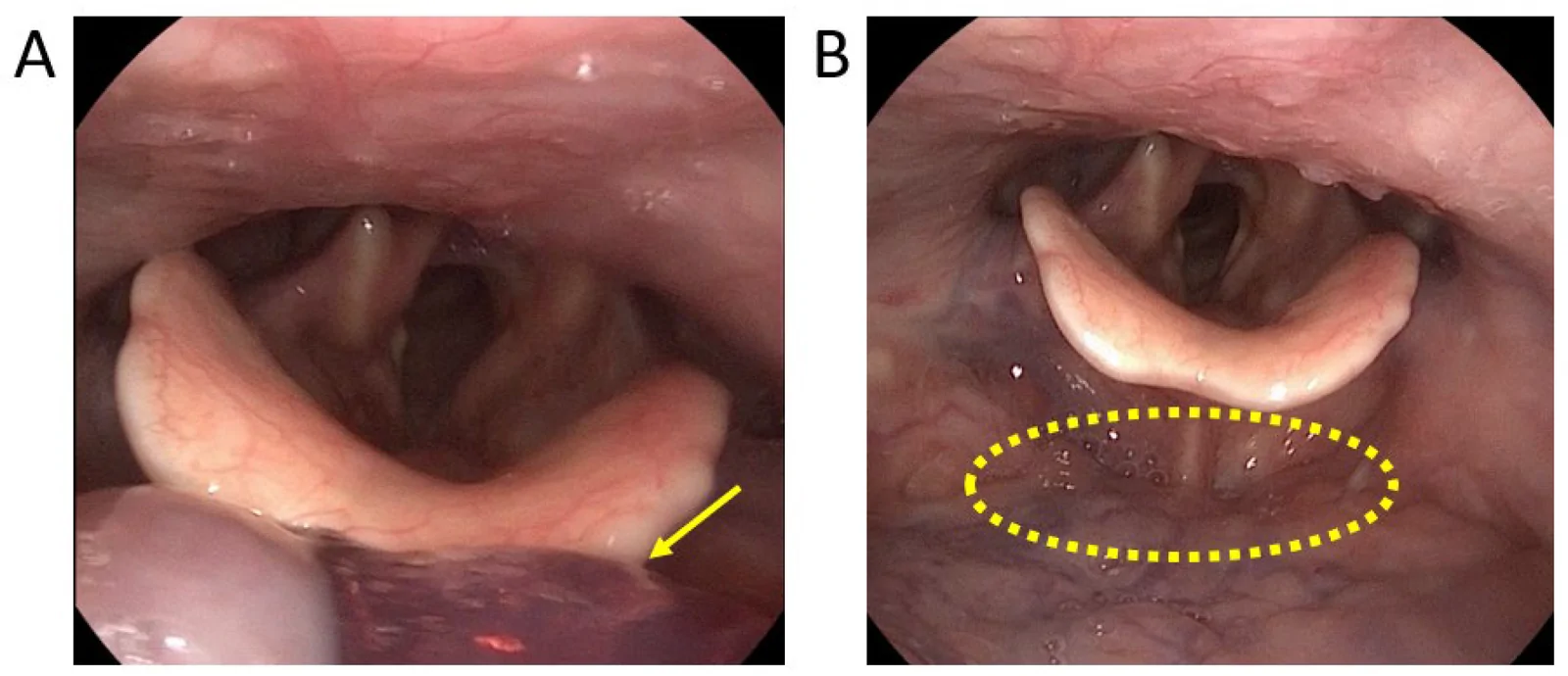
Fiberoptic endoscopic swallowing examination of the alginate beads prepared by direct spherification with an 8 min residence time: (**A**) initiation of swallowing—the content in the right vallecula and on the epiglottis—showing delayed initiation of swallowing (marked with a yellow arrow); (**B**) after swallowing—no residue in the valleculae or piriform sinuses after swallowing (marked by a yellow-outlined area).

After thorough evaluation of different alginate hydrogel beads’ texture, rheology, and stability, one was selected to ensure the product’s key qualities: containing at least 95% water; meeting Level 6 soft and bite-sized foods standards per IDDSI; forming an elastic membrane that is tough to break with fingers but dissolves easily in the mouth; and remaining stable for at least one week during storage. An alginate bead with an 8 min residence time was selected for this experiment because of its favorable hardness, low weight loss, and ease of handling. Although the bead with the shortest residence time had the lowest hardness and weight loss—potentially making it ideal for the test—its hardness was too low to be comfortably grasped by fingers. As a result, a slightly firmer alginate bead (8 min), which also exhibited textural and rheological properties compatible with safe swallowing, was chosen for the study. During swallowing, delayed initiation was observed; the chewed bolus fell onto the epiglottis. There was no full “white window” during swallowing, and there were no signs of aspiration (Figure 5A). Based on the Yale Pharyngeal Residue Severity Rating Scale (YPRSRS) (Figure 5B), the bolus was completely swallowed, with no residue remaining in the valleculae or piriform sinuses. The patient reported no discomfort while swallowing and found the hydrogel beads easy to consume. Based on the 8-point Penetration–Aspiration Scale (PAS), the risk of aspiration during consumption of the sample was rated 1 point: no penetration or aspiration, with contents not reaching near the vocal cords.

Preliminary FEES results reinforcing the conclusions drawn from the rheological, textural, and IDDSI tests imply the suitability of the developed formulation for patients with mild-to-moderate dysphagia; however, further research is needed.

#### 2.2.3. Vitamin Stability During Storage

Another potential application of these hydrogel beads is the delivery and delayed release of vitamins, as discussed in this section. Encapsulation efficiencies for vitamins B9 and B12 were 58% and 45%, respectively, regardless of residence time. This contradicts the results of other authors [47], which indicate that both CaCl_2_ concentration and CaCl_2_ curing bath time affect the encapsulation efficiency of different bioactives in the alginate structures. It was also reported that sodium alginate hydrogel beads with varying concentrations of γ-polyglutamic acid achieved vitamin B9 encapsulation efficiencies ranging from 29.16% to 83.87% [48]. The low vitamin encapsulation efficiency is likely due to the production method used. Small vitamin molecules in their unbound form probably diffuse out of the hydrogel matrix into the cross-linking solution during preparation, a process further enhanced by the large surface area of the forming hydrogel, resulting in faster diffusion [49]. To increase vitamin encapsulation, it would likely be necessary either to encapsulate the vitamins separately before incorporating them into the three-dimensional alginate network or to further reinforce the alginate network using biopolymer blends, coatings, etc.

**Figure 6:**
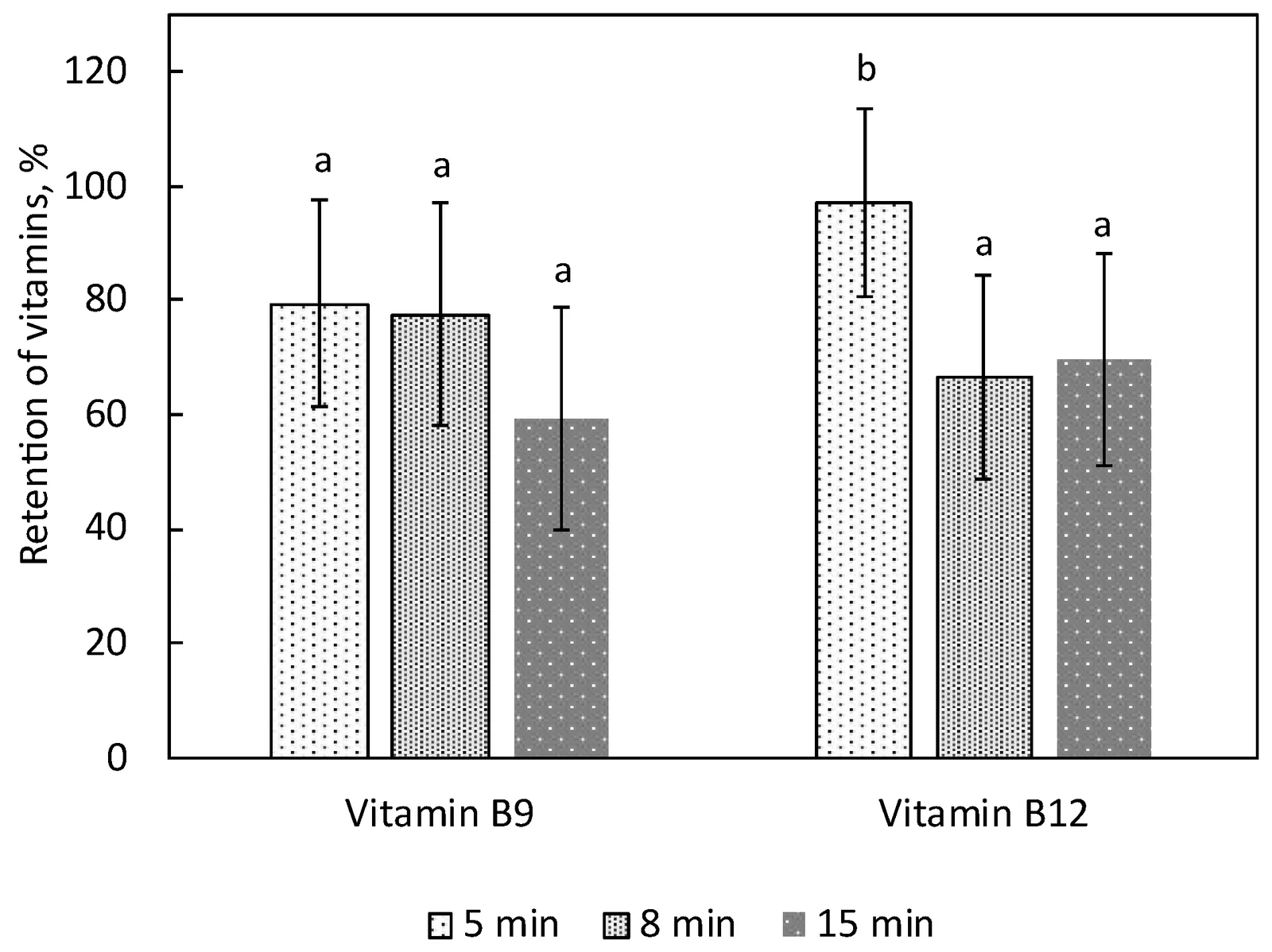
Stability of vitamins B9 and B12 after 6 weeks of storage in alginate hydrogel beads produced by frozen direct spheroidization at varying residence times (*n* = 3). (a, b) Different superscript letters indicate significant (*p* < 0.05) differences in vitamin retention among alginate hydrogel beads with different residence times.

Figure 6 shows the stability of these vitamins after 6 weeks of storage. Samples with the shortest residence time of 5 min retained higher vitamin levels, with 79.4% for vitamin B9 and 97% for vitamin B12 after 6 weeks. This pattern is more noticeable for vitamin B12. Longer residence times led to decreased stability, with remaining vitamin levels around 60% to 70% after storage, which is still quite good given the duration. This may be due to increased weight loss (see Table 1), as well as cross-linking density changes, which accelerate vitamin leakage from the alginate matrix. It is believed that during storage, intermolecular interaction strength is reduced, which could negatively affect vitamin loading capacity. Consistent results were observed for phenolic compounds and vitamin C encapsulated in the liquid-core alginate beads, with a significant decrease over the 7-day storage period, attributed to oxidation and degradation within the liquid core [19].

#### 2.2.4. Vitamins Released During *In Vitro* Digestion

The release behavior of the encapsulated bead with an 8 min residence time was evaluated under simulated gastric and intestinal conditions that mimic the digestive process in older adults. This sample was selected based on fiberoptic endoscopic evaluation; it was confirmed to be safe to swallow, whereas the sample with the shorter residence time, despite higher vitamin stability, was too soft to handle and transfer to the mouth by hand. The control sample was a non-gelled aqueous solution of the vitamin under test.

**Figure 7:**
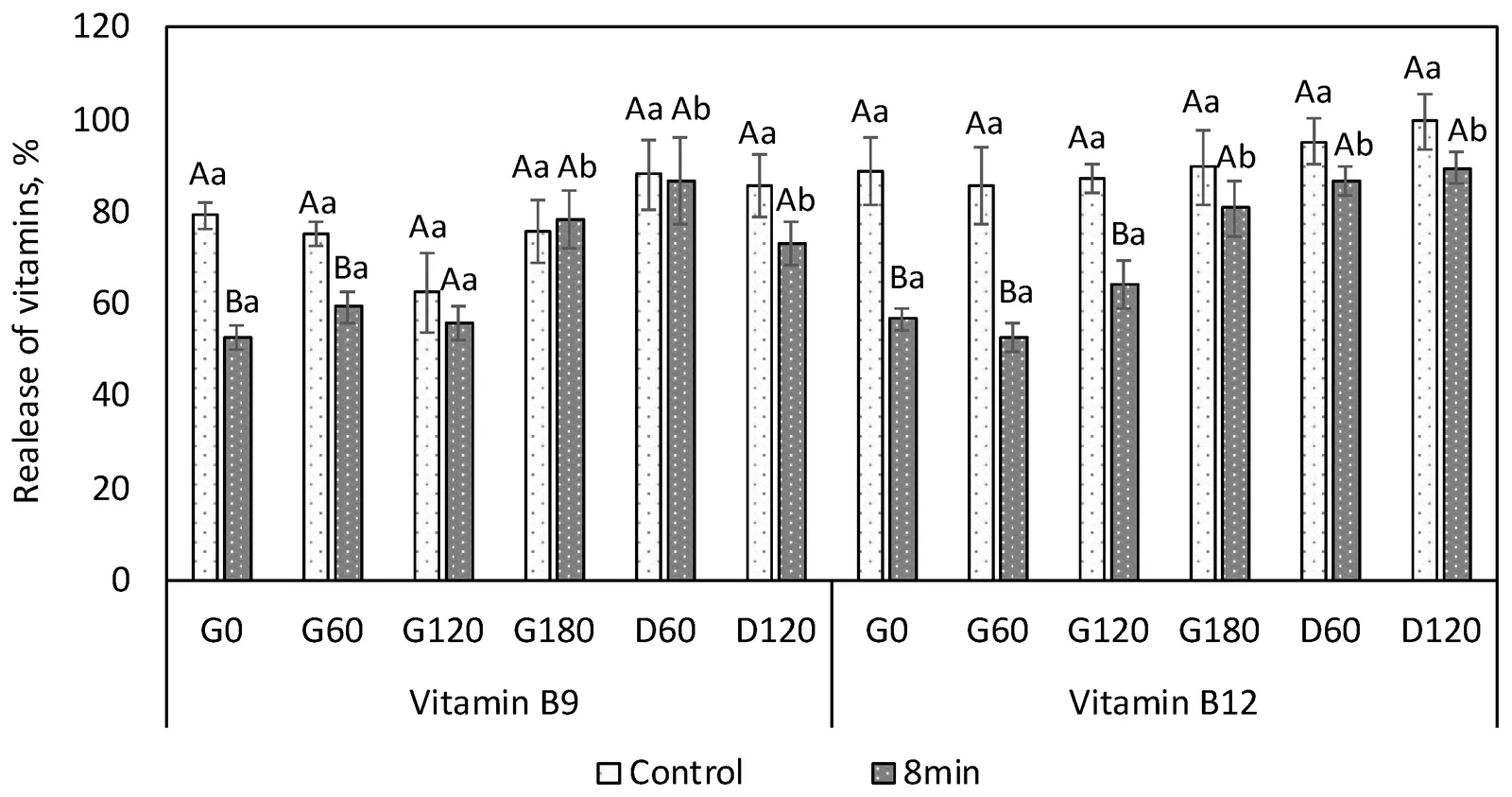
Release of vitamins B9 and B12 during in vitro digestion in alginate hydrogel beads produced by frozen direct spheroidization with an 8 min residence time (*n* = 2). G0—gastric phase 0 min; G60—gastric phase 60 min; G120—gastric phase 120 min; G180—gastric phase 180 min; D60—intestinal phase 60 min; D120—intestinal phase 120 min. (a, b) Different superscript letters indicate significant (*p* < 0.05) differences in vitamin release during in vitro digestion. (A, B) Different superscript letters indicate significant (*p* < 0.05) differences in vitamin release among alginate beads and controls. Control—water solution of vitamin B9 or B12, respectively.

Under gastric conditions, the structure formed in the alginate beads delays the release of vitamins B9 and B12 relative to the control but only during the first 120 min of the gastric stage. At the end of the gastric phase (at G180), the alginate beads released a significant amount of encapsulated vitamins—78.6% for vitamin B9 and 80.8% for vitamin B12—closely matching the control vitamin solution, which showed 75.9% and 89.7%, respectively (Figure 7). Such excessive release in this part of the intestine is undesirable and is assumed to be related to the size of the encapsulated molecules, the alginate network mesh size, and swelling-disintegration (erosion) [50]. At low pH, ion exchange between calcium and sodium from digestive fluid is accelerated, resulting in extensive swelling and disintegration of the alginate beads [51]. As previously reported, small molecules such as vitamins are released at acidic pH, and a rise in pH slightly accelerates the process [49]. Furthermore, a larger mesh size may accelerate the diffusion of low-molecular-weight vitamins, similar to digestive enzymes, leading to quicker disintegration of the alginate network [50,51]. Nevertheless, some authors reported minimal folic acid release from alginate-γ-polyglutamic acid hydrogel beads under similar conditions. The discrepancy is due to a more resilient, tightly packed alginate hydrogel network, reinforced by interactions between alginate and γ-polyglutamic acid [48]. Such a stronger, interpenetrating hydrogel network also slowed the release of vitamin B12 from alginate hydrogel beads enhanced with various protein sources [52], as well as from vitamin B12-loaded core-shell hydrogel beads coated or filled with inulin [53]. In our case, a more densely packed bead network with a firmer texture might pose a limitation, possibly rendering the beads unsuitable for a dysphagia diet. Nonetheless, future studies could investigate enhanced vitamin protection by refining the alginate bead formulation without drastically affecting the beads’ textural, swallow-related characteristics.

Under simulated intestinal conditions, the beads initially released approximately 10% more encapsulated vitamins, with a final release of 86.9–89.6% (Figure 7). At a higher pH, deprotonation of carboxyl groups to COO- increases the polymer’s negative charge. This electrostatic repulsion causes the matrix to swell, facilitating the release of the encapsulated compound [54]. More importantly, vitamins within the bead structure exhibited the same release characteristics as those in the non-gelled solution. This indicates that additional structural modifications, such as multilayer coatings, mixed biopolymers, or composite hydrogels, are necessary to enhance targeted intestinal delivery without compromising swallowing safety.

## 3. Conclusions

This study demonstrates that frozen direct spherification is an effective method for creating high-water-content alginate structures with customized textures, enabling hydration and safe swallowing while maintaining satisfactory retention of vitamins B9 and B12 during storage. Conversely, reverse-spherified liquid-core beads were deemed unsuitable for dysphagia due to structural instability during oral processing. Residence time was identified as a crucial factor affecting bead microstructure, mechanical properties, and stability: increasing the exposure time of alginate to calcium ions increased hardness but also increased weight loss and reduced vitamin stability during 6 weeks of storage. Although alginate encapsulation delayed vitamin release during the initial stage of gastric digestion, it did not provide sustained protection throughout the gastric phase, indicating limited control over vitamin delivery at the specific site. Future studies should optimize the hydrogel bead formulation to enhance gastric protection and targeted intestinal delivery without compromising swallowing safety and evaluate the acceptability of sensory characteristics and comfort of consumption among older adults.

The present study is also limited to evaluating the developed hydrogel beads in a single patient as a proof-of-concept case study. Although the findings provide preliminary evidence of feasibility, further research involving larger patient cohorts with various etiologies and severities of dysphagia is necessary to confirm the reproducibility, safety, and clinical utility of the proposed formulations.

## 4. Materials and Methods

### 4.1. Materials

Sodium alginate powder (E-401) Algin, trisodium citrate (E-331iii) Citras, a commercial mixture of calcium chlorate (94–97%, E-509) and potassium chlorate (E-508) Calcic, as well as a commercial blend of calcium lactate (E-327) and calcium gluconate (E-578) Gluco, were sourced from Texturas Ferran (Adria, Barcelona, Spain). Glycerol (C_3_H_8_O_3_), CAS No. 56-81-5, was supplied by Labochema LT (Vilnius, Lithuania). The cherry–apple juice concentrate (containing 3.1% cherry juice concentrate and 3.1% apple juice concentrate) was purchased from “Purena”, Belfood (Złoty Potok, Poland). Citric acid was obtained from “UAB Klingai” (Kaunas, Lithuania). Sodium benzoate and potassium were also obtained from Eurochemicals (Vilnius, Lithuania). Vitamins B9 and B12 were supplied by DSM Nutritional Products Ltd. (Basel, Switzerland).

### 4.2. Preparation of Alginate Hydrogel Beads by the Direct Spherification Method

Sodium alginate (1% *w*/*w*), glycerol (1% *w*/*w*), potassium sorbate (0.1% *w*/*w*), and sodium benzoate (0.05% *w*/*w*) were weighed and mixed with a water (71–78% by mass) and fruit juice concentrate (18–27% by mass) mixture. The solution was homogenized with a Philips hand blender (600 W HR1364) at maximum speed for 2 min at room temperature. The pH was measured with a Milwaukee MW102 PRO+ pH meter (Szeged, Hungary) and adjusted to 3.9–4.0 with 2 M trisodium citrate. The solution was placed in an Ultrasonic Cleaner Proclean 3.0 DSP (Ulsonix, Ulange, Shenzhen, China) for 8 min at 30 kHz. It was then poured into hemispherical molds in ~5 g portions and frozen at −44 °C for 2 h. A 1% solution of calcium chlorate and potassium chlorate was prepared by dissolving the mixture of salts in distilled water.

A cross-linking procedure was performed as follows: the frozen hydrogel solutions were transferred to a bath containing a 1% mixture of calcium chlorate and potassium chlorate at room temperature (20 ± 1 °C). The hydrogel beads were cross-linked for 5, 8, or 15 min. After cross-linking, they were removed and briefly immersed in a water bath for 1–2 min. Finally, the hydrogel beads were wiped dry with a paper towel and stored in covered containers at +4°C for 42 days.

### 4.3. Preparation of Alginate Hydrogel Beads by the Reverse Spherification Method

A mixture comprising 5% calcium lactate and calcium gluconate, along with 5% potassium sorbate and 0.1% sodium benzoate, was weighed and added to a water (71–78% *w*/*w*) and fruit juice concentrate (18–27% *w*/*w*) mixture. The solution was then homogenized with a Philips hand blender (600 W HR1364) at maximum speed for 2 min at room temperature. The solution was poured into hemispherical molds in approximately 5 g portions and frozen for 2 h at −44 °C. Additionally, a 0.5% sodium alginate solution was prepared using distilled water and homogenized for 2 min at room temperature with the same blender.

The cross-linking process involved transferring the frozen hydrogel beads into a 0.5% sodium alginate bath for 5, 8, or 15 min at room temperature (20 ± 1 °C). Afterward, they were taken out and rinsed in a water bath for 1–2 min. Finally, the hydrogel beads were stored in juice-filled plastic containers at +4 °C for 42 days. This approach aimed to prevent osmotic pressure differences from affecting the beads’ properties.

In a later stage, to assess the effectiveness of these alginate hydrogel beads for vitamin encapsulation and delivery, hydrogel beads were prepared with vitamins B9 (0.0006% *w*/*w*) and B12 (0.004% *w*/*w*) by following all the previously described procedures, incorporating these vitamins into the alginate solution as appropriate.

### 4.4. Characterization of Differently Prepared Alginate Hydrogel Beads

#### 4.4.1. Texture Profile Analysis (TPA)

Texture profile analysis was performed using a TA.XT Plus texture analyzer (Stable Micro Systems Ltd., Godalming, UK). The test involved compressing the sample at 1 mm/s to 50% of its height using a P/20 aluminum cylinder, followed by a 5 s pause between compressions. Force–time data were used to calculate the material hardness, cohesiveness, and adhesiveness.

#### 4.4.2. pH

The pH of the alginate beads was measured directly at 20 ± 2 °C using a digital pH meter (Milwaukee MW102 PRO+, Hungary) equipped with an N1048A penetration probe.

#### 4.4.3. Weight Loss

Weight loss was measured by comparing the hydrogel beads’ initial mass after production to their mass at a specific point during storage. The hydrogels, prepared via reverse spherification and stored in water, were removed, carefully dried with paper towels, and weighed. Weight loss was calculated as follows:

(1) $$weight\;loss\;(\%)=\frac{\left(m_{i}\;-\;m_{t}\right)}{m_{i}}\cdot 100;$$

where *m_i_*—initial mass of the hydrogel bead, g; *m_t_*—mass of the hydrogel bead at a specific time during storage, g.

#### 4.4.4. Contact Angle

To evaluate the sphericity of the alginate hydrogel beads obtained, the contact angle was measured according to procedures reported in the literature [55]. The contact angle of the hemispherical hydrogel beads was determined from photographs captured with a high-resolution digital camera. These images were analyzed using ImageJ software 1.53 t with the Contact Angle Plug-in (National Institutes of Health, Bethesda, MD, USA) to obtain θ, which was then used to calculate the contact angle. 

#### 4.4.5. International Dysphagia Diet Standardisation Initiative (IDDSI) Tests

The compliance of the obtained hydrogel beads for the dysphagia diet was evaluated using IDDSI testing methods, specifically employing fork pressure and fork separation tests.

#### 4.4.6. Swallow-Related Rheological Properties

Rheological properties were assessed at 25 °C with a plate-to-plate configuration (20 mm diameter, 1 mm gap) with a Physica MCR92 rheometer (Anton Paar, Graz, Austria).

The viscosity was determined at a steady shear rate of 50 s^−1^ and 25 °C, following the classification standards of the National Dysphagia Diet Task Force (NDD) [56].

Small Amplitude Oscillatory Shear (SAOS) was used to evaluate the viscoelastic behavior within the linear viscoelastic (LVE) region. The storage (G′) and loss (G′′) moduli were recorded across an angular frequency range from 0.1 to 100 rad s^−1^.

A Large Amplitude Oscillatory Shear (LAOS) test involved sweeping the oscillation amplitude from 0.01% to 1000% strain at a constant 1 Hz frequency to assess how the storage modulus (G′), loss modulus (G′′), and loss tangent (tan δ) depend on strain.

The shear recovery behavior was assessed via a three-step flow sweep test, comprising three shear rate holds: 0.1 s^−1^ for three minutes, 50 s^−1^ for one minute, and 0.1 s^−1^ for three minutes.

A flow sweep over shear rates from 0.001 to 0.1 s^−1^ was applied to evaluate the shear yield stress. The shear yield stress was identified as the initial peak in shear stress.

#### 4.4.7. Fiberoptic Endoscopic Evaluation of Swallowing (FEES)—Case Study

The Fiberoptic Endoscopic Evaluation of Swallowing was performed at the Geriatrics Center of LSMU Kaunas Hospital. The study adhered to the Declaration of Helsinki and received approval from the Kaunas Regional Biomedical Research Ethics Committee (protocol code P1-BE-2-59/2018, dated 11 October 2022). All patients provided informed consent. A 78-year-old patient was assessed and diagnosed with mild to moderate oropharyngeal dysphagia according to the Dysphagia Outcome and Severity Scale (DOSS). The patient was found to be at high risk of aspiration (8-point Penetration–Aspiration Scale (PAS), 6 points) when drinking unmodified liquids and at low risk (PAS, 3 points) with modified liquids. The specific cause of the dysphagia remains unidentified.

The FEES procedure used a 3.7 mm HD Video Rhino-Laryngoscope (STORZ, Bestdata, Tuttlingen, Germany), following the FEES Examination Protocol (Langmore, 2000) [57]. Aspiration risk was assessed with the Penetration–Aspiration Scale (PAS), pharyngeal residue was scored with the Yale Pharyngeal Residue Severity Rating Scale (YPRSRS), and oropharyngeal dysphagia severity was scored with the Doss. Fluids were colored with dyes (Rainbow Dust, Preston, UK). Patients were examined with water and thickened fluids, followed by alginate hydrogel beads.

#### 4.4.8. Determination of Vitamins B9 and B12

Encapsulated vitamins B9 and B12 were quantified by high-performance liquid chromatography (HPLC). Prior to analysis, alginate hydrogel beads were mechanically crushed, mixed with an equal weight of distilled water to obtain a homogeneous suspension, and filtered through a 0.22 μm syringe membrane filter. Chromatographic analysis was performed on a Nexera-i LC-2040C 3D Plus HPLC system (Shimadzu, Kyoto, Japan) fitted with an Atlantis dC18 analytical column (5 μm particle size, 4.6 × 150 mm; Waters, Milford, MA, USA). Separation was achieved with a binary mobile phase consisting of 0.1% trifluoroacetic acid (TFA) in water (eluent A) and 0.1% TFA in acetonitrile (eluent B). The gradient elution program was as follows: 0–5 min, 0–3% B; 5–6 min, 3–15% B; 6–10 min, 15–20% B; 10–12 min, 20–100% B; followed by an isocratic hold at 100% B until 25 min. The mobile phase was delivered at a constant flow rate of 1.4 mL min^−1^, with a 20 μL injection volume, and the column was thermostatically maintained at 30 °C. Eluting compounds were monitored at 280 nm using a diode-array detector. The analytical method provided limits of detection (LOD) of 0.20 and 0.10 for vitamins B9 and B12, respectively, and the corresponding limits of quantification (LOQ) were 0.70 and 0.30. Calibration curves demonstrated excellent linearity, with a coefficient of determination (R^2^) of 0.998. Retention of vitamins during the processing was calculated using the following equation:

(2) $$Retention=(\frac{C_{in}\;-\;C_{f}}{C_{in}})\times 100\%;$$

where *C_in_* represents the initial amount of vitamin added to the hydrogel bead, and *C_f_* indicates the amount of vitamin measured after storage at 4 °C for 6 weeks.

#### 4.4.9. Vitamin Release During *In Vitro* Digestion

The gastrointestinal fate of the hydrogel beads was evaluated using a static *in vitro* digestion model based on the INFOGEST protocol for older adults [58,59]. Before digestion, the beads were compressed to 50% of their original height using a texture analyzer to simulate the mechanical forces generated during chewing. An aqueous vitamin solution containing the same amounts of vitamins B9 and B12 as the hydrogel beads was digested under identical conditions and served as the control. For the oral phase, 5.0 g of sample was combined with 2.00 g of glass beads and 5 mL of simulated salivary fluid containing α-amylase (75 U/mL). After 2 min of oral processing, 10 mL of simulated gastric fluid supplemented with pepsin (1200 U/mL) was added, and the total volume was adjusted to 20 mL. Gastric digestion was carried out for 180 min at 37 °C in a shaking water bath (Thermolab, GFL 1092, Berlin, Germany) operating at 140 rpm. The intestinal stage was followed immediately by adding simulated intestinal fluid containing pancreatin (80 U/mL) and bile salts to achieve a final bile salt concentration of 6.7 mmol/L. Digestion continued for an additional 120 min, increasing the total digest volume to 40 mL. Throughout the experiment, the gastric phase was maintained at approximately pH 3.7, whereas the intestinal phase was conducted at pH 6.5–7.0. pH changes were recorded at the start of gastric digestion (G0), after 60, 120, and 180 min of the gastric stage (G60, G120, and G180), and after 60 and 120 min of the intestinal stage (D60 and D120). To terminate gastric proteolysis, the digest was neutralized to pH 7.0 with 6 M NaOH, thereby inactivating pepsin. Upon completion of digestion, samples were rapidly cooled in an ice-water bath (0–4 °C), centrifuged at 4000 rpm and 4 °C (MPW-260R, MPW Med. Instruments, Warsaw, Poland), and the recovered supernatants were passed through a filter, frozen, and stored at −18 °C until further analysis. All digestion experiments were performed in duplicate. The concentrations of vitamins released into the gastrointestinal fluids were quantified by HPLC as described above, and the percentage of vitamin release was calculated using the following equation:

(3) $$Release=(\frac{C_{f}}{C_{in}})\times 100\%$$

where *C_in_* denotes the vitamin amount in the hydrogel beads, and *C_f_* represents the vitamin amount measured in digesta.

### 4.5. Statistical Analysis

All experiments were performed a minimum of three repetitions (*n* = 3), with results expressed as mean ± standard deviation. Statistically significant differences among means were determined with a *p*-value of less than 0.05 via analysis of variance (ANOVA) conducted using Statistica 12.0 (StatSoft Inc., Tulsa, OK, USA, 2013). The mean values were compared through Duncan’s multiple-range test.

## Figures and Tables

**Table 1:** pH and weight loss of the alginate hydrogel beads prepared by frozen direct and reverse spheroidization with different residence times during 42 days of storage.

Residence Time, min	Storage Duration, days						
	0	7	14	21	28	35	42
	*Direct Spherification*						
	pH						
5	3.97 ± 0.03 ^aD^	4.06 ± 0.01 ^bE^	4.06 ± 0.02 ^cE^	3.98 ± 0.02 ^cD^	3.89 ± 0.05 ^bC^	3.81 ± 0.02 ^bB^	3.77 ± 0.02 ^aA^
8	4.00 ± 0.04 ^aBC^	3.98 ± 0.00 ^aB^	3.97 ± 0.03 ^bB^	3.95 ± 0.03 ^bB^	3.83 ± 0.01 ^abA^	3.83 ± 0.01 ^bA^	3.82 ± 0.03 ^bA^
15	3.90 ± 0.01 ^aD^	3.89 ± 0.01 ^aD^	3.88 ± 0.02 ^aD^	3.84 ± 0.02 ^aC^	3.83 ± 0.04 ^abC^	3.77 ± 0.01 ^aB^	3.72 ± 0.01 ^aA^
	Weight loss, %						
5	-	2.13 ± 0.18 ^aA^	4.69 ± 0.51 ^aB^	7.33 ± 0.74 ^aC^	10.08 ± 1.35 ^aD^	13.09 ± 1.73 ^aE^	15.69 ± 1.37 ^aF^
8	-	2.73 ± 0.54 ^aA^	7.97 ± 1.01 ^bB^	11.25 ± 1.08 ^bC^	14.41 ± 1.20 ^bD^	17.02 ± 1.94 ^bE^	19.29 ± 1.57 ^bF^
15	-	6.44 ± 0.52 ^bA^	13.20 ± 0.81 ^cB^	17.09 ± 0.89 ^cC^	20.37 ± 1.29 ^cD^	23.27 ± 0.49 ^cE^	25.24 ± 0.65 ^cF^
	*Reverse spherification*						
	pH						
5	3.94 ± 0.02 ^aAB^	3.92 ± 0.02 ^aA^	3.91 ± 0.02 ^aA^	4.04 ± 0.00 ^aC^	4.07 ± 0.03 ^bD^	4.05 ± 0.02 ^aC^	4.01 ± 0.02 ^bB^
8	3.94 ± 0.02 ^aA^	3.93 ± 0.02 ^aA^	3.96 ± 0.01 ^bB^	4.04 ± 0.01 ^aD^	4.02 ± 0.02 ^abCD^	4.06 ± 0.03 ^aD^	4.00 ± 0.02 ^bC^
15	3.91 ± 0.01 ^aA^	3.97 ± 0.03 ^bB^	3.99 ± 0.03 ^bcBC^	4.05 ± 0.01 ^aC^	3.97 ± 0.01 ^aB^	4.05 ± 0.01 ^aC^	3.95 ± 0.02 ^aB^
	Weight loss, %						
5	-	4.27 ± 1.80 ^aA^	6.34 ± 2.01 ^bB^	7.64 ± 1.15 ^bC^	8.96 ± 1.71 ^aD^	9.68 ± 1.51 ^bE^	10.41 ± 1.88 ^bF^
8	-	6.66 ± 0.66 ^cA^	7.47 ± 1.66 ^cB^	8.85 ± 1.20 ^cC^	10.65 ± 1.70 ^bD^	10.14 ± 1.70 ^cD^	11.57 ± 0.95 ^cE^
15	-	5.19 ± 1.30 ^bA^	5.70 ± 1.00 ^aAB^	7.23 ± 1.02 ^aBC^	8.36 ± 0.59 ^aCD^	8.34 ± 0.86 ^aCD^	9.69 ± 0.93 ^aE^

Values are reported as means ± standard deviation (*n* = 3); data labeled with different lowercase letters (a–c) show statistically significant differences (*p* < 0.05) between alginate hydrogel beads with different residence times; data labeled with different uppercase letters (A–F) show statistically significant differences (*p* < 0.05) during storage time.

**Table 2:** Textural properties of alginate hydrogel beads prepared by frozen direct and reverse spheroidization with different residence times.

Residence Time, min	Hardness, g	Adhesiveness, g·s	Cohesiveness
*Direct spherification*			
5	77.48 ± 1.18 ^a^	−10.42 ± 0.35 ^a^	0.35 ± 0.05 ^b^
8	105.07 ± 22.14 ^b^	−11.85 ± 3.26 ^a^	0.29 ± 0.08 ^ab^
15	136.25 ± 8.88 ^c^	−11.14 ± 3.53 ^a^	0.29 ± 0.01 ^ab^
*Reverse spherification*			
5	27.21 ± 0.60 ^a^	-	-
8	34.87 ± 0.13 ^b^	-	-
15	62.89 ± 0.35 ^c^	-	-

Values are reported as means ± standard deviation (*n* = 3); different letter superscripts (a–c) represent significant (*p* < 0.05) differences in textural properties between alginate hydrogel beads with different residence times.

## Data Availability

Data are contained within the article.
